# The first solid-state route to luminescent Au(I)—glutathionate and its pH-controlled transformation into ultrasmall oligomeric Au_10–12_(SG)_10–12_ nanoclusters for application in cancer radiotheraphy

**DOI:** 10.3389/fchem.2023.1178225

**Published:** 2023-06-05

**Authors:** Andrea Deák, Pál T. Szabó, Vendula Bednaříková, Jaroslav Cihlář, Attila Demeter, Michaela Remešová, Evelina Colacino, Ladislav Čelko

**Affiliations:** ^1^ Supramolecular Chemistry Research Group, Institute of Materials and Environmental Chemistry, Research Centre for Natural Sciences, Budapest, Hungary; ^2^ Centre for Structure Study, Research Centre for Natural Sciences, Budapest, Hungary; ^3^ High-Performance Materials and Coatings for Industry Research Group, Central European Institute of Technology, Brno University of Technology, Brno, Czechia; ^4^ Renewable Energy Research Group, Institute of Materials and Environmental Chemistry, Research Centre for Natural Sciences, Budapest, Hungary; ^5^ ICGM, Univ Montpellier, CNRS, ENSCM, Montpellier, France

**Keywords:** bioactive molecules, gold nanocluster, gold thiolate, glutathione, mechanochemistry

## Abstract

There is still a need for synthetic approaches that are much faster, easier to scale up, more robust and efficient for generating gold(I)–thiolates that can be easily converted into gold–thiolate nanoclusters. Mechanochemical methods can offer significantly reduced reaction times, increased yields and straightforward recovery of the product, compared to the solution-based reactions. For the first time, a new simple, rapid and efficient mechanochemical redox method in a ball-mill was developed to produce the highly luminescent, pH-responsive Au(I)–glutathionate, [Au(SG)]_*n*_. The efficient productivity of the mechanochemical redox reaction afforded orange luminescent [Au(SG)]_*n*_ in isolable amounts (mg scale), usually not achieved by more conventional methods in solution. Then, ultrasmall oligomeric Au_10–12_(SG)_10–12_ nanoclusters were prepared by pH-triggered dissociation of [Au(SG)]_*n*_. The pH-stimulated dissociation of the Au(I)–glutathionate complex provides a time-efficient synthesis of oligomeric Au_10–12_(SG)_10–12_ nanoclusters, it avoids high-temperature heating or the addition of harmful reducing agent (e.g., carbon monoxide). Therefore, we present herein a new and eco-friendly methodology to access oligomeric glutathione-based gold nanoclusters, already finding applications in biomedical field as efficient radiosensitizers in cancer radiotherapy.

## 1 Introduction

**FIGURE 1:**
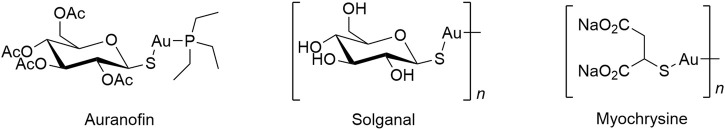
Gold drugs used in the treatment of rheumatoid arthritis.

Gold complexes have attracted extensive interest in their research because of their unique composition and structures, intriguing optical properties, as well as, a wide variety of applications, including catalysis, chemical sensing, biomedical imaging and cancer treatment (Jin et al., 2011). Over the years, a family of gold(I)-thiolates including oligomers and polymers, thiolate-protected gold nanoclusters (AuNCs) and nanoparticles (AuNPs) with unique optical properties such as luminescence or surface plasmon resonance were successfully synthesized (Chakraborty and Pradeep, 2017). The glutathionate-coated AuNCs often display selective and high accumulation in cancerous tissues (Zhang et al., 2014a; Zhang et al., 2014b; Zhang et al., 2015; Du et al., 2017) and many of them were used as efficient radiosensitizers in cancer radiotherapy (Zhang et al., 2014a; Zhang et al., 2014b; Zhang et al., 2015). Some Au(I)-thiolate complexes, such as gold(I) thiopyranosate (Auranofin), gold(I) thioglucose (Solganal) and gold(I) sodium thiomalate (Myochrysine) were widely used as therapeutic agents for the treatment of rheumatoid arthritis (Figure 1) (Shaw, 1999).

**FIGURE 2:**
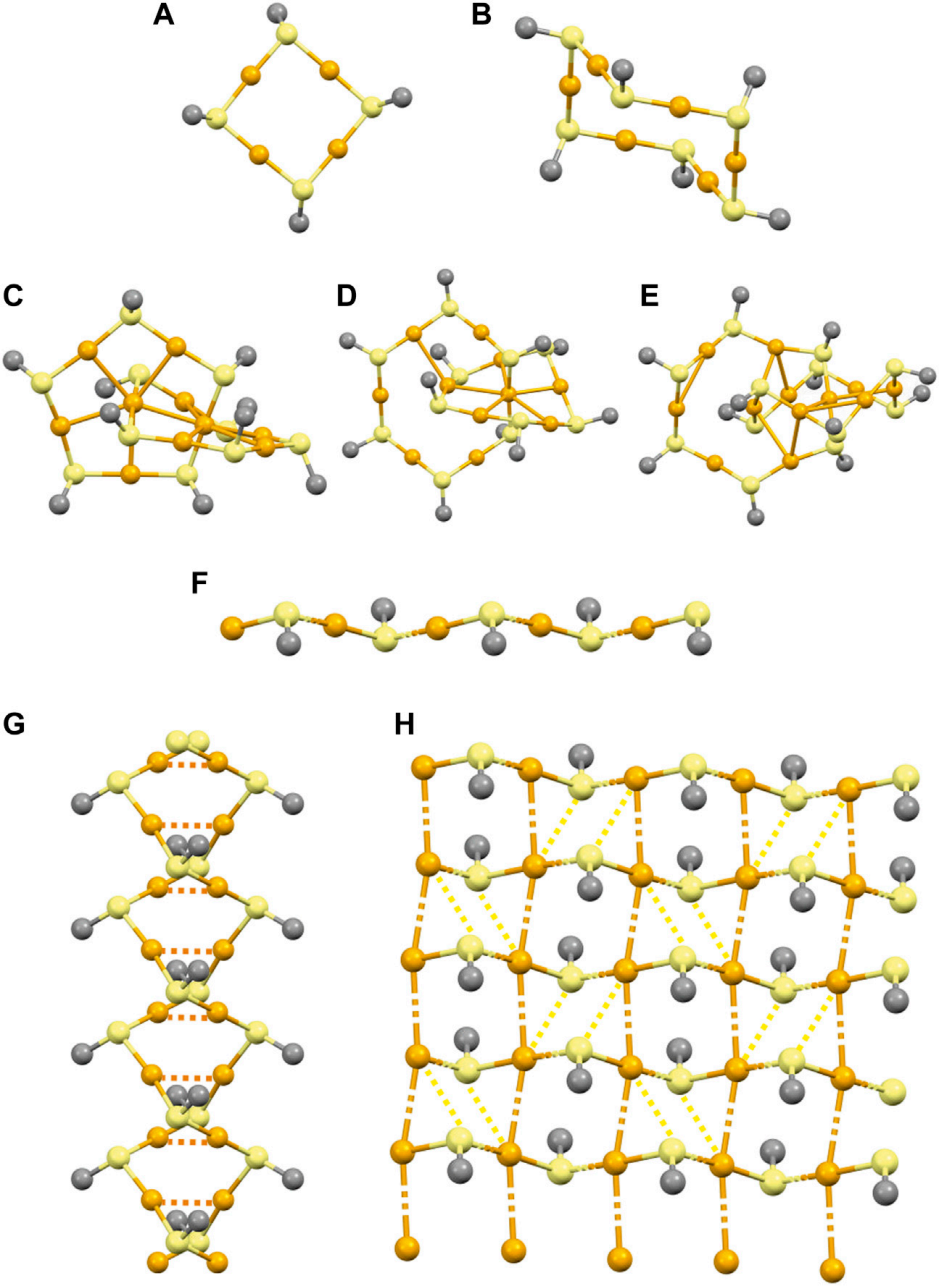
Possible structures of gold(I)-thiolate oligomers [**(A–B)**: Rings and **(C–E)**: Catenanes] and CPs **(F)**. Self-assembly of Au(I)–thiolate CPs into **(G)** double interpenetrated helical chains and **(H)** 2D lamellar structure. Color code: Au: orange; S: yellow; R group: grey.

Despite the importance of gold(I)-thiolates in medicinal and material chemistry, as well as, nanosciences, only a few were structurally characterized because of their prevalent poor solubility and/or poor crystallinity. Gold(I)-thiolates with cyclic oligomeric or extended coordination polymeric (CP) structures (Figure 2) were reported (Bonasia et al., 1993; Bau, 1998; Wiseman et al., 2000; Chui et al., 2006; Schröter and Strähle, 2006; Lavenn et al., 2015; Lavenn et al., 2016; Veselska et al., 2017; Veselska et al., 2019). Gold(I)-thiolate [Au(SR)]_*n*_ oligomers were observed to form either rings [*n* = 4 and 6; (Bonasia et al., 1993; Schröter and Strähle, 2006); Figures 2A, B] or interlocked rings, catenane [*n* = 10–12; (Wiseman et al., 2000; Chui et al., 2006); Figures 2C–E] structures. The gold(I)-thiolate CPs (Figure 2F) can often self-assemble into double interpenetrated helical chains (Bau, 1998; Lavenn et al., 2015) (Figure 2G) or lamellar 2D structures (Lavenn et al., 2016; Veselska et al., 2017; Veselska et al., 2019) (Figure 2H) through a large set of aurophilic Au(I)···Au(I) and/or additional Au(I)–S interactions. Gold(I)-thiolates were investigated both for their unique structural features (Lavenn et al., 2015; Lavenn et al., 2016) as well as for their morphological transformations (Nie et al., 2013; Nie et al., 2014; Dai et al., 2019). In addition to the above mentioned non-covalent interactions, such as aurophilic and secondary Au–S, the self-assembly of Au(I)-thiolates having pH-sensitive carboxylic ligand function can also be driven by hydrogen bonding interactions. At lower pH values, the carboxylate functions participate in hydrogen bonding interactions, which promote the formation of aurophilic Au(I)···Au(I) and further Au(I)–S interactions leading to the assembly of Au(I)–thiolate CPs into lamellar structures (Nie et al., 2014). At higher pH, the repulsion between the deprotonated carboxylate groups is strong, which can weaken the aurophilic and secondary Au(I)–S interactions, hindering the assembly of Au(I)–thiolate CPs into 2D structures. Thus, higher pH values can favor the formation of unassembled or partially assembled nanostructures (Nie et al., 2014). These reversible and dynamic aggregation–dissociation (assembly-disassembly) morphological transformations of Au(I)-thiolates having pH-sensitive carboxylic ligand functions can be controlled by changing the pH (Nie et al., 2013; Nie et al., 2014).

Gold(I)-thiolates are usually prepared by hydrothermal synthesis in autoclave, by mixing HAuCl_4_ with an excess of thiol to reduce Au(III) into Au(I) coupled with the oxidation of thiol to disulfide (Lavenn et al., 2015; Veselska et al., 2017; Veselska et al., 2019). This hydrothermal method requires high temperature (120 or 150°C) and long reaction time (18 or 24 h) (Lavenn et al., 2015; Veselska et al., 2017; Veselska et al., 2019), however, relatively milder synthetic conditions, lower temperatures (60 or 80°C) also coupled with long reaction time (18 or 48 h) (Lavenn et al., 2016; Vaidya et al., 2020) were used to obtain gold(I)-thiolates. Moreover, thiolate-protected AuNCs or AuNPs can be prepared by the reduction or reductive decomposition of Au(I)-thiolates (Zhou et al., 2010; Luo et al., 2012; Wu et al., 2020). They can also be obtained by the reaction between an Au(III) salt, thiol (HSR) and a strong reducing agent, such as sodium borohydride (Negishi et al., 2004; Negishi et al., 2005; Shichibu et al., 2007; Briñas et al., 2008; Wu et al., 2009a; Wu et al., 2009b), superhydride (Corbierre and Lennox, 2005) or carbon monoxide (Yu et al., 2013; Yu et al., 2014; Wu et al., 2020). Au(I)–glutathionate oligomers, CPs and NCs were also obtained from glutathione (GSH = γ-Glu-Cys-Gly), a natural thiol-containing tripeptide, frequently used as a reducing agent (Zhou et al., 2010; Luo et al., 2012; Wu et al., 2019; Wu et al., 2020). Owing to the presence of carboxylic and amino groups of the glutathionate ligand, the Au(I)–glutathionate [Au(SG)]_*n*_, displays pH-sensitive behavior in water. (Odriozola et al., 2007; Briñas et al., 2008; Luo et al., 2012). Hainfeld and co-workers showed that the gold(I)–glutathionate CPs can adopt different sizes depending on the pH of solution (Briñas et al., 2008). Lower pH favors the formation of larger polymers (Briñas et al., 2008), while higher pH values favor smaller polymeric structures (Briñas et al., 2008). Xie and co-workers reported that the dissolution of insoluble [Au(SG)]_*n*_ polymer (by addition of NaOH 1 M) leads to its oligomerization (Luo et al., 2012; Zhang et al., 2014b). Thus, oligomeric Au(I)-glutathionates with a well-defined molecular formula such as the concomitantly formed Au_10_(SG)_10_, Au_11_(SG)_11_ and Au_12_(SG)_12_ nanoclusters (with zero confined electrons through the cluster Au(*n*) core, meaning that only Au(I) species are present in these structures, differently that a classical Au_*n*_(SG)_*m*_ gold cluster, usually containing a core of *n* number of Au (0) atoms that share confined electrons) can be obtained at neutral pH in the absence (Zhang et al., 2014b) or in the presence of carbon monoxide (Yu et al., 2013; Du et al., 2017; Wu et al., 2020). Only one report described the production (and isolation) of oligomeric Au_10–12_(SG)_10–12_ nanoclusters under pH-controlled reaction conditions in the presence of aqueous solution (12.5 mL, 20 mM) of HAuCl_4_ (98.5 mg were used), GSH and gaseous (and harmful) carbon monoxide as reducing agent (Yu et al., 2013). However, the amount of the collected dispersions (∼100 mg) most likely refers to the quantity of starting Au(III) salt and not to the amount of the resulting AuNCs. Oligomeric AuNCs such as Au_10–12_(SG)_10–12_ finds application in the medical field as radiosensitizers showing ultrahigh tumor targeting specificity and uptake, good biocompatibility (and low toxicity), combined with an efficient renal clearance, being also able to enhance the therapeutic efficiency of radiotherapy (Zhang et al., 2014b; Du et al., 2017). Therefore, there is a need to develop efficient synthetic methods to access them in isolable amounts.

The water insoluble Au(I)–glutathionate [Au(SG)]_*n*_ is precursor for the glutathione-protected AuNCs, and it is usually prepared in diluted (typically millimolar concentration, e.g., 1.1, 20 or 50 mM) aqueous solutions (Schaaff et al., 1998; Luo et al., 2012; Wu et al., 2019). These reactions generally require a relatively long reaction time (24 h) (Wu et al., 2019) and yield sols (Luo et al., 2012; Wu et al., 2019) or cloudy suspensions hampering its isolation (Schaaff et al., 1998), with any possibility to determine a yield of the process. Additionally, the throughput of the reaction (scale-up) can lead to reproducibility problems and may induce changes in the composition or aggregation. Therefore, the development of more sustainable synthetic methods to access Au(I)–thiolates is of great interest, not only to increase the productivity of the targets (higher throughput per unit of time because of faster reaction kinetics), but also for providing more robust and efficient methods to scale Au(I)–thiolates up. In this regard, their mechanochemical preparation from “*highly concentrated solid solutions*” proved to be extremely beneficial leading to quantitative yield of water insoluble Au(I)–thiolate in short reaction time, also allowing its straightforward isolation.

Indeed, the synthesis, isolation and purification of many Au(I)-thiolates and thiolate-protected AuNCs can be very challenging or even impossible to achieve in conventional solution-based methods. Mechanochemical methods (James et al., 2012; Howard et al., 2018; Tan and Garcia, 2019; Friščić et al., 2020; Porcheddu et al., 2020; Virieux et al., 2021) are sustainable, (Ardila-Fierro and Hernández, 2021; Colacino et al., 2021; Fantozzi et al., 2022; Galant et al., 2022; Sharma et al., 2022), answer the needs for faster reaction kinetics (Mulas et al., 2010; Colacino et al., 2018; Carta et al., 2020; Crawford et al., 2020), higher productivity (Crawford et al., 2020) and scalability (Colacino et al., 2021), also challenging transformations impossible to be achieved using solution-based methods (Cuccu et al., 2022). Their use to access value-add monomers for polymer industry [e.g., benzoxazines (Martina et al., 2018), e-caprolactame (Mocci et al., 2021; Baier et al., 2022), *N*-chloro hydantoins (Konnert et al., 2016) etc], including active pharmaceutical ingredients (Colacino et al., 2019; Pérez-Venegas and Juaristi, 2020; Ying et al., 2021) APIs is also reported.

In spite of their advantages, mechanochemical methods have only rarely been used for the synthesis of gold(I) compounds (Do et al., 2018; Ingner et al., 2020) and gold-based nanostructures (Rak et al., 2014; de Oliveira et al., 2020b) or for the creation of new polymorphic forms (Seki et al., 2015; Seki et al., 2016; Yagai et al., 2016; Jin et al., 2017; Vainauskas et al., 2023). We previously reported the mechanochemical synthesis of stimuli-responsive nanosized (Deák, 2019) mononuclear Au (diphos)X (X = Cl or I) (Baranyai et al., 2015; Deák et al., 2021) and dinuclear [Au_2_ (diphos)_2_](X)_2_ (X = NO_3_, BF_4_, PF_6_ or SbF_6_) complexes (Jobbágy et al., 2014; Deák et al., 2015; Jobbágy et al., 2016) of diverse diphosphine (diphos) ligands as well as dicyanoaurate-based heterometallic CPs (Jobbágy et al., 2011). Recently, Camargo and co-workers reported the mechanochemical synthesis of AuNPs with targeted sizes and shapes by using gold(III) or gold(I) salts, stabilizing agents (polyvinylpyrrolidone) and reductants (sodium borohydride, ascorbic acid or hydroquinone) (de Oliveira et al., 2019; de Oliveira et al., 2020a). Prasad and co-workers prepared thiolate-protected gold nanoclusters by the solventless solid state grinding of gold(I) octanethiolate with sodium borohydride (Bera et al., 2018). Mechanochemical protocols, were however successfully applied only to the production of thiolate-protected silver nanoclusters (Rao et al., 2010).

## 2 Materials and methods

### 2.1 Reagents and materials

All chemicals and solvents used for the syntheses were of reagent grade. The solvents for synthesis were used without further purification.

### 2.2 Synthesis

HAuCl_4_ 3H_2_O (78.8 mg, 0.2 mmol; finely homogenized in an agate mortar) and the glutathione (GSH) (307 mg, 1 mmol) ligand (1:5 ratio) were added into a 10 mL agate milling jar. After adding two 10 mm diameter agate balls (weight of each ball m = 1.5 g, m_*tot*_ = 2 × 1.5 g), the reaction mixture was ball milled for 40 min in a Retsch MM400 mill at 25 Hz. The resulting orange-emitting product was scrapped out from the jar and then it was suspended in water (30 mL), which allowed the removal of any ligand excess, oxidized glutathione disulfide (GSSG) by-product. The white [Au(SG)]_*n*_ precipitate (51.4 mg; yield = 51%) was isolated by filtration and washed with water. Then, the oligomeric Au_10–12_(SG)_10–12_ nanoclusters were prepared by the dissolution of water-insoluble [Au(SG)]_*n*_ in a minimum amount of aqueous NaOH (0.5 M) solution (till pH ∼7). The formation of Au_10–12_(SG)_10–12_ oligomers in the resulting clear and colorless solution was confirmed by ESI-MS(−) analyses. These oligomeric Au(I)-thiolate NCs can be precipitated by ethanol addition, and after centrifugation the pellet can be redissolved in water.

### 2.3 Characterization methods

The X-ray diffractometry was performed by Rigaku SmartLab 3 kW (Rigaku, Japan). The diffractometer was set up in Bragg-Brentano geometry using Cu Kα radiation (λ = 1.54 Å) in a range of 1°–80° at a scanning speed of 1.5°/min and operated at 40 kV and 30 mA. The X-ray photoelectron spectroscopy (XPS) measurements were performed with a Kratos Axis Supra spectrometer (Manchester, United Kingdom), with a monochromatic Al Kα X-ray source (1486.69 eV), an emission current of 15 mA, a hybrid lens mode and a charge neutralizer. A wide and high-resolution spectrum was recorded with a pass energy of 80 eV and 20 eV, using scanning steps of 1.0 and 0.1 eV, respectively. XPS spectra were analyzed using CasaXPS software version 2.3.17PR1.1. The obtained spectra were calibrated using C 1s peaks with a fixed value of 284.8 eV. Varian Scimitar 2000 FT-IR spectrometer (Varian Inc., United States) equipped with broad band MCT (mercury-cadmium-telluride) detector and “Golden Gate” ATR (attenuated total reflection) diamond accessory. The FT-IR spectra were collected at nominal resolution of 4 cm^–1^ by co-addition of 64 individual scan. Thermogravimetric analysis (TGA), differential scanning calorimetry (DSC), and coupled mass spectrometry (MS) were carried out simultaneously (TGA-DSC-MS) using a Netzsch STA 409c/CD apparatus. Analyzes were performed on the as-obtained materials in an argon atmosphere (100 mL Ar/min) using Al_2_O_3_ crucibles. Heating from 30°C to 750°C at a rate of 5°C/min and cooling from 750°C to 100°C at a rate of 20°C/min were used. The morphology of the samples was examined with a scanning electron microscope (SEM, Mira3, Tescan, Czech Republic) and a high-resolution SEM (HRSEM, Verios 460 L, FEI, Czech Republic) in secondary electron (SE) and back-scattered electron (BSE) mode using an acceleration voltage of 5 and 10 kV. Prior to the SEM observations, the samples were coated with a 14 nm thick carbon layer using an evaporation coating unit (EM ACE600, Leica, Germany). The corrected luminescence spectra were recorded using a JASCO FP-8300 spectrofluorometer with a 5 nm resolution and a 355 nm excitation. The excitation spectrua was recorded at the maximum of the emission (635 ± 10 nm). Phosphorescence decays were detected with a 500 MHz Tektronix TDS 640A oscilloscope (635 ± 10 nm), excited by 355 nm flashes of an Nd-YAG laser (Continuum Surelite) at a very low light intensity (less than 0.03 mJ per flash). Averaged 50–100 flashes were fitted with two- or three-exponential decay models (OriginPro 2018). The absolute emission quantum yield of the solid sample of [Au(SG)]_*n*_ was determined on a Jobin-Yvon Fluoromax-4 spectrofluorometer equipped with a Ø2″ integrating sphere (Thorlabs). The fluorescent quantum yield (Φ) of the aggregated particles was measured relative to the 9,10-bis(phenylethynyl)anthracene standard (Φ = 0.99). (Demeter, 2014). The UV-Vis spectra were recorded on an UNICAM UV500 spectrophotometer. ESI-MS measurements were performed on a Sciex TripleTOF 5600+ high-resolution mass spectrometer equipped with a Duospray ion source (combined electrospray and atmospheric pressure chemical ionization). The resolution was at least 25,000 over the entire mass range (900–3,000 Da). The sample solution was flow injected into a 0.2 mL/min mobile phase (acetonitrile:water 50:50). The mass spectrometer was operated in a negative electrospray mode. Spectra were collected in MCA mode. Analyst TF 1.7.1. software was used for controlling the measurements and PeakView 2.2.0. with BioTools add-on was used for data processing and deconvolution. DLS measurement was performed using a W130i apparatus (Avid Nano Ltd., United Kingdom) and using a low volume disposable cuvette (UVette, Eppendorf Austria GmbH, Austria).

## 3 Results and discussion

**SCHEME 1:**
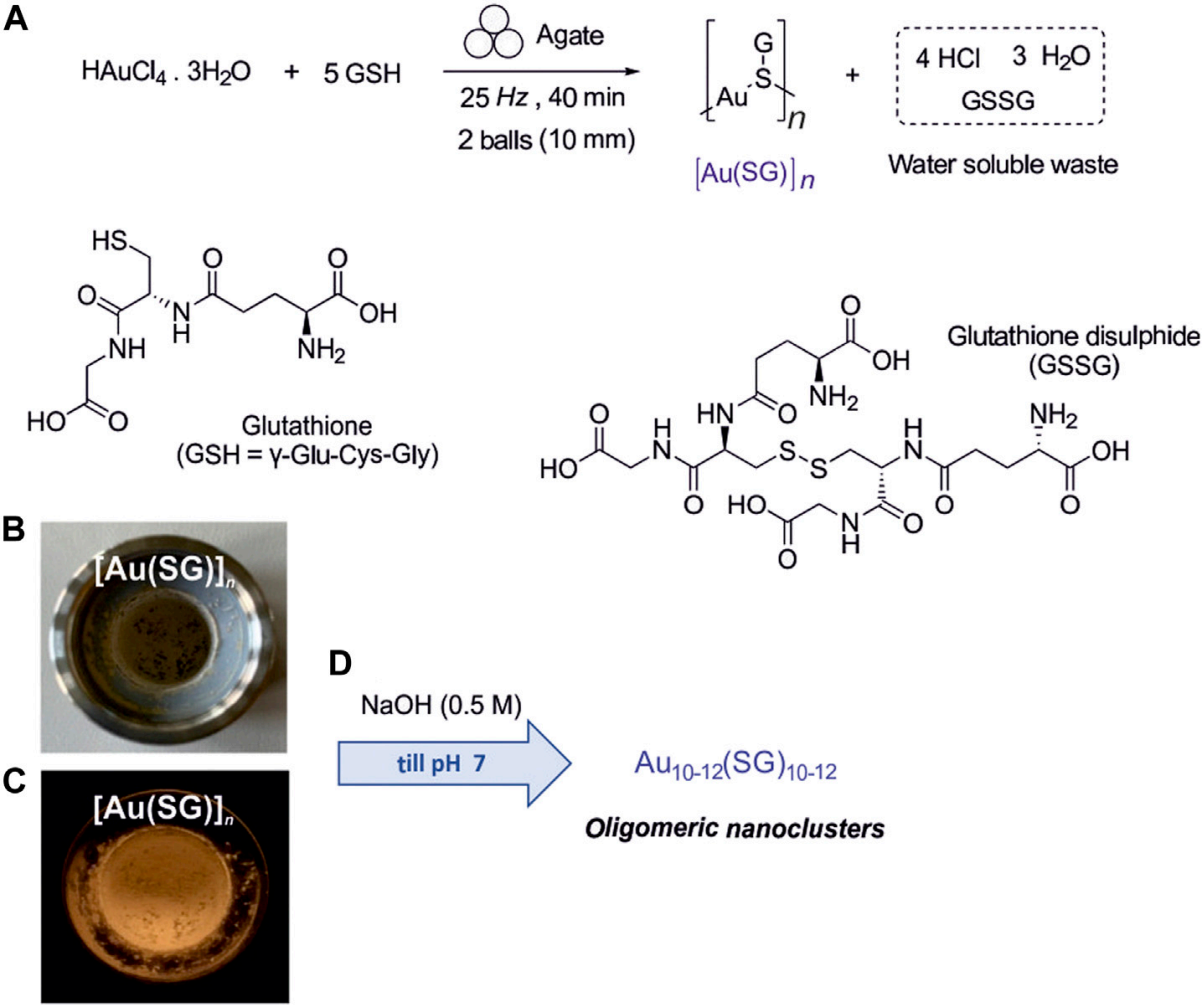
**(A)** Reductive mechanochemical synthesis of [Au(SG)]_*n*_. Photographs of the so-formed [Au(SG)]_*n*_ were taken under **(B)** ambient light and **(C)** 365 nm UV lamp illumination. **(D)** pH-controlled formation of oligomeric Au_10–12_(SG)_10–12_ nanoclusters.

We demonstrate mechanochemistry for direct, room-temperature reductive conversion of a gold (III) precursor by GSH into a [Au(SG)]_*n*_ complex, followed by the pH-controlled formation of discrete oligomeric Au_10–12_(SG)_10–12_ nanoclusters (Scheme 1).

We developed a new simple and efficient mechanochemical approach for the synthesis of Au(I)–glutathionate (Scheme 1a), which enables its rapid formation and in a higher amount (51.4 mg) compared to the previously reported solution-based methods (yielding not isolable sols or cloudy suspensions). Therefore, the reactants were ball-milled for 40 min, followed by the dispersion of the reaction mixture in water. In the first step, the reductive conversion of the gold (III) precursor tetrachloroauric acid trihydrate (HAuCl_4_ 3H_2_O) occurred in the ball-mill in the presence of glutatione (GSH) as a reducing and stabilizing agent. The reduction of Au(III) to Au(I) was evidenced by the change of the reaction mixture color from dark yellow to white (Scheme 1b), and by the appearance of an intense orange luminescence (Scheme 1c) after 40 min of ball-milling. The so-formed powder was dispersed in water, and the highly luminescent, orange-emitting [Au(SG)]_*n*_ complex precipitated out of the solution. At the same time, the GSH used in excess, the unreacted HAuCl_4_ (in insignificant amounts) and the glutathion disulfide by-product remained in the aqueous phase and could be filtered off (Scheme 1a). In contrast to previously reported solution-based methods carried out in diluted media yielding suspensions at low concentration (Schaaff et al., 1998; Luo et al., 2012; Wu et al., 2019), the [Au(SG)]_*n*_ was obtained in a significant amount (>50 mg) when prepared by mechanochemistry. This water-insoluble [Au(SG)]_*n*_ complex with a bright orange luminescence was characterized by PXRD, SEM, XPS, FT-IR, STA and emission spectroscopy.

**FIGURE 3:**
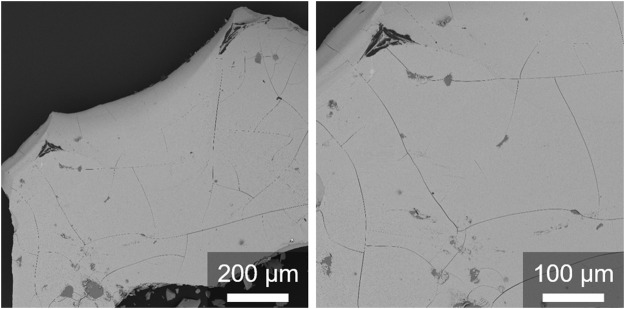
SEM-BSE micrographs of the [Au(SG)]_*n*_ complex.

The PXRD pattern shows the amorphous (Supplementary Figure S1) nature of the bright orange luminescent powder of [Au(SG)]_*n*_. Its insolubility supports the formation of extended coordination polymeric (Schaaff et al., 1998; Zhang et al., 2014b) instead of soluble oligomeric structures (*n* = 10–12) (Odriozola et al., 2007). Scanning electron microscopy (SEM) images (Figure 3) for amorphous [Au(SG)]_*n*_ show large irregular particles with a flat surface morphology covered with nearly invisible cracks.

**FIGURE 4:**
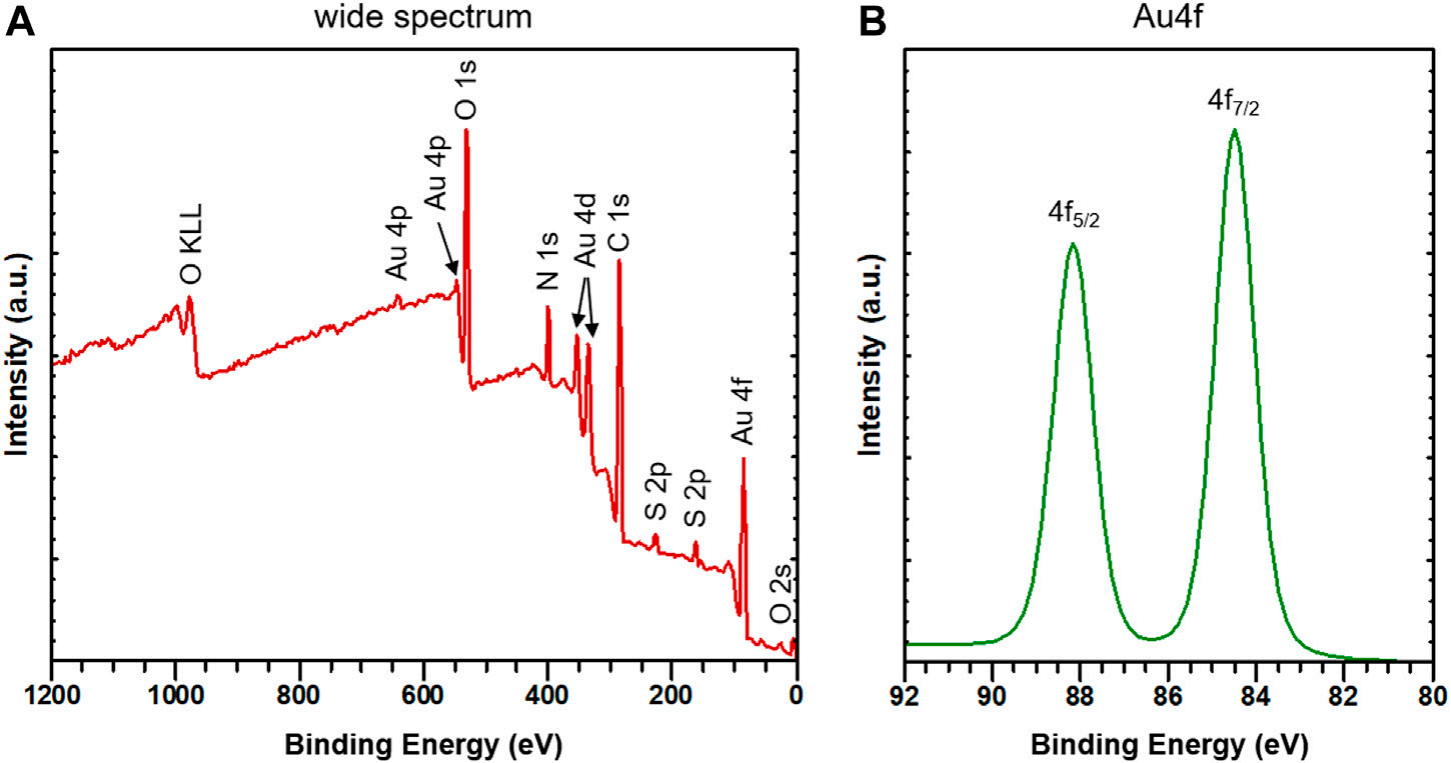
XPS spectra **(A)** wide spectrum and **(B)** high-resolution spectrum of Au4f. XPS spectrum shows the Au(4f) binding energy of the as-synthesized orange-emitting [Au(SG)]_*n*_ complex.

The reduction of Au(III) into Au(I) and the formation of the white-colored [Au(SG)]_*n*_ complex was confirmed by X-ray photoelectron spectroscopy (XPS). As shown in Figure 4A, the XPS spectrum shows the characteristic peaks for each element (except H, which cannot be detected by lab-based equipment) present in [Au(SG)]_*n*_. The Au 4f XPS spectrum (Figure 4B) shows the binding energy of Au 4f_5/2_ and Au 4f_7/2_ at 88.2 eV and 84.5 eV, respectively. This is practically identical to that reported previously for polymeric Au(I) glutathionate, where the Au 4f_5/2_ and Au 4f_7/2_ binding energies are at 88.1 eV and 84.4 eV, respectively (Luo et al., 2012). The position and the sharpness of these peaks with full width at half maximum of 0.88 and 0.89 eV indicate that all gold ions are in +1 oxidation state (Lavenn et al., 2015). The white color of the prepared [Au(SG)]_*n*_ complex also confirms the presence of Au(I) and the absence of Au (0) atoms. In large gold (0) nanocrystals, the Au 4f_5/2_ and Au 4f_7/2_ binding energies are at 87.3 eV and 83.8 eV, respectively (Luo et al., 2012). The FT-IR spectroscopy shows that the characteristic S–H stretching vibration (2,523 cm^–1^) of the free GSH ligand completely disappeared from the spectrum of the [Au(SG)]_*n*_ complex (Supplementary Figure S2), which confirms the coordination of the thiolate function of the tripeptide to Au(I).

Also, simultaneous thermal analysis (STA), thermogravimetric analysis (TGA), differential scanning calorimetry (DSC) coupled with mass spectrometry (MS) on the [Au(SG)]_*n*_ complex were performed. The TGA shows that the thermal decomposition of the thiolate ligands coordinated to Au(I) started at around 200°C (Supplementary Figure S3). The Au/SG ratio calculated from the weight loss of the glutathionate ligand is 1/1.1, and this is in good agreement with the expected value (for the details, see the Supplementary Material).

**FIGURE 5:**
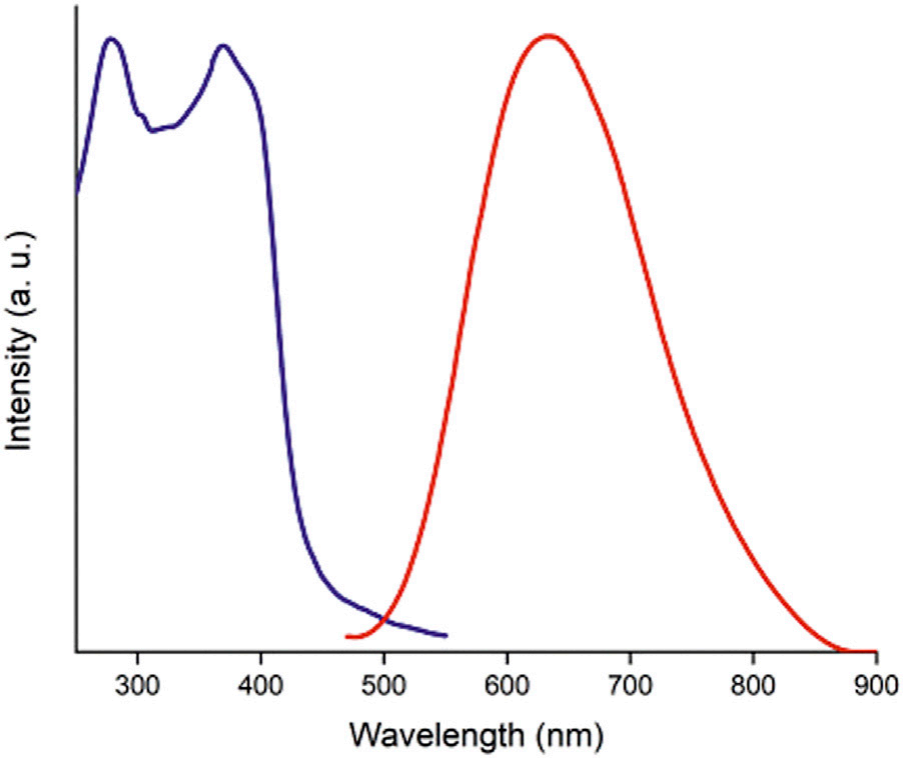
Solid state corrected photoluminescence (red line) and excitation (blue line) spectra of [Au(SG)]_*n*_.

As shown in Figure 5, the solid-state emission spectrum (λ_ex_ = 355 nm) of orange-emitting [Au(SG)]_*n*_ complex displays a broad band with an emission maximum centered at 635 nm. There are two maxima at 280 and 370 nm in the excitation spectrum (λ_em_ = 635 nm, Figure 5). The emission lifetime in the microsecond timescale [2.59 µs (52%) and 0.49 µs (48%)] is similar to that of Au(I)-glutathionate prepared in aqueous media at low pH (Wu et al., 2019). The fluorescence quantum yield was 0.037 ± 0.08 when measured at 355 nm excitation. Control measurement was also performed relative to zona-refined pyrene crystals (Φ = 0.68) (Katoh et al., 2009), which were consistent with the data reported above.

**FIGURE 6:**
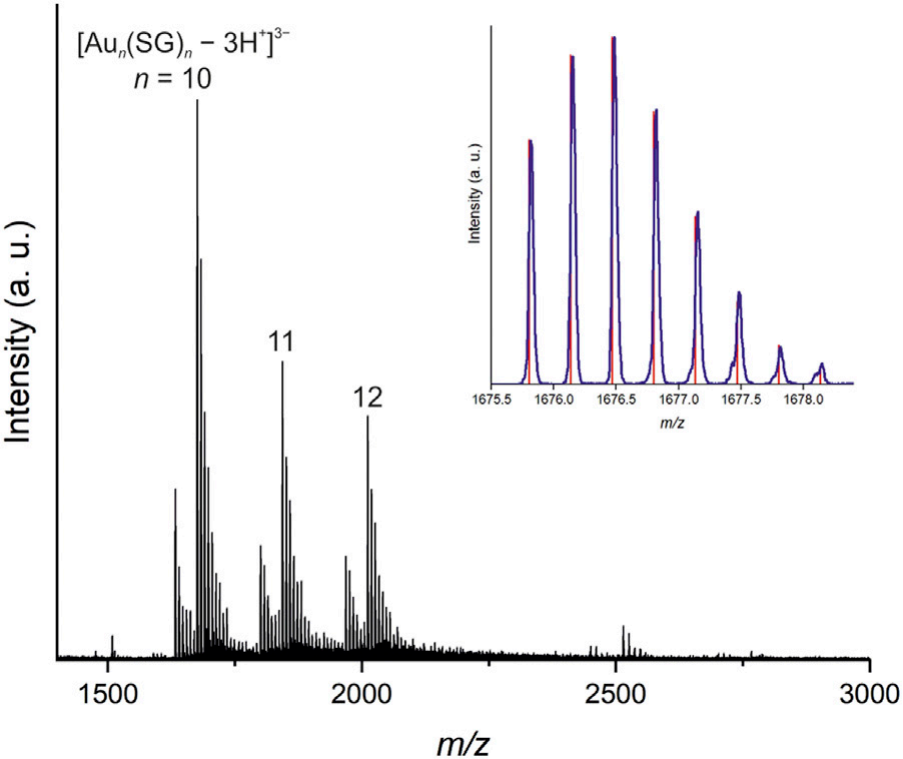
Negative ion mode ESI MS spectrum of oligomeric Au_10–12_(SG)_10–12_ nanoclusters formed upon pH-controlled (∼7) dissociation of [Au(SG)]_*n*_. The inset shows the experimental (blue) and simulated (red) isotope patterns of [Au_10_(SG)_10_—3H^+^]^3–^.

We further investigated the dissociation behavior of the water insoluble gold(I)–glutathionate, and this material was dissolved in a minimal amount of NaOH (0.5 M) by adjusting the pH to ∼ 7 till dissolution of the sample. The negative ion mode ESI-MS spectrum shown in Figure 6, indicates the formation of Au_10–12_(SG)_10–12_ upon pH-controlled dissociation of [Au(SG)]_*n*_ (Scheme 1). As shown in Figure 6, three intense peaks at 1675.8178, 1843.5006, and 2011.1834 mass/charge ratio (*m/z*) values are observed, which can be readily assigned to [Au_10_(SG)_10_—3H^+^]^3*–*^ [Au_11_(SG)_11_—3H^+^]^3–^ and [Au_12_(SG)_12_—3H^+^]^3–^ cluster ions carrying a triple negative charge. These assignments were confirmed by an isotope pattern analysis, which shows that the respective experimental and simulated isotope patterns of these ions are in perfect agreement (Figure 6 inset, Supplementary Figures S5, S6). The ESI MS analysis indicates the coexistence of [Au_10_(SG)_10_—3H^+^]^3*–*^ [Au_11_(SG)_11_—3H^+^]^3–^ and [Au_12_(SG)_12_—3H^+^]^3–^ cluster ions in a relative ratio of 51%, 27%, and 22%. Other sets of peaks can be assigned to [Au_10_(SG)_10_—(2 + *n*)H + *n*Na]^2–^ (*n* = 0–5; Supplementary Figure S7) [Au_10_(SG)_10_—(3 + *n*)H + *n*Na]^3–^ (*n* = 0–9; Supplementary Figure S8) [Au_11_(SG)_11_—(3 + *n*)H + *n*Na]^3–^ (*n* = 0–9; Supplementary Figure S9), and [Au_12_(SG)_12_—(3 + *n*)H + *n*Na]^3–^ (*n* = 0–9; Supplementary Figure S10) in the ESI-MS(−) mass spectrum. The UV-Vis absorption spectrum (see Supplementary Figure S11) also confirm the formation of the oligomeric nanocluster, displaying a characteristic absorption at 310 nm (shoulder) for Au_10–12_(SG)_10–12_ species (Bertorelle et al., 2017).

**FIGURE 7:**
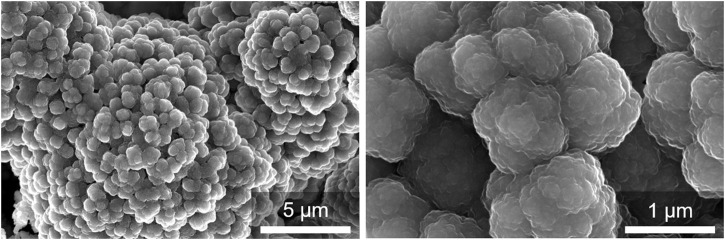
HRSEM-SE micrographs of the aggregated small particles of oligomeric Au_10–12_(SG)_10–12_ nanoclusters resulted from the pH-controlled dissociation of [Au(SG)]_*n*_.

HRSEM-SE images were recorded to further confirm the pH-controlled dissociation of the [Au(SG)]_*n*_ structure. HRSEM-SE micrographs (Figure 7) illustrated that a change in the pH (∼ 7) triggered the dissociation of bulky irregular particles of [Au(SG)]_*n*_ (Figure 3) into spherical particles. These ethanol-aggregated small particles with an average diameter between 1 μm and 150 nm stack together and form aggregates without any specific morphology (Figure 7). The hydrodynamic diameter and the polydispersity index (PDI) of these particles resulted from the pH-controlled dissociation of [Au(SG)]_*n*_ followed by their ethanol-induced aggregation were measured by DLS. This analysis showed that the average hydrodynamic diameter of aggregated particles was 179.4 nm (Supplementary Figure S12) with PDI value of 1.20. Thus, the solution containing the gold(I)–glutathionate oligomers was clear and practically nonluminescent, but turned cloudy and luminescent upon addition of ethanol owing to the formation of aggregates. The emission maximum of these aggregated gold(I)–glutathionate oligomers is centered at 647 nm (λ_excit_ = 355 nm, Supplementary Figure S13), and displays three-exponential decay with lifetimes of 0.20 µs (35%), 1.1 µs (23%), and 4.60 µs (42%), respectively. The fluorescence quantum yield was 0.06 ± 0.01 at 280 nm excitation, and 0.04 ± 0.01 at 380 nm excitation (Demeter, 2014).

## 4 Conclusion

This study showed that mechanochemistry can be used for a simple, rapid and efficient synthesis of Au(I)–glutathionate that exhibits a bright orange luminescence. To the best of our knowledge, this is the first report on the mechanochemical reduction of Au(III) into Au(I) with an excess of thiol to form a gold(I) thiolate in a short reaction time at room temperature. The mechanochemical method herein reported to prepare Au(I)-glutathionate outperformed compared to solution-based procedures developed for gold(I)–thiolates because it occurs: i) In shorter reaction times (40 min instead of 18–48 h for solution-based methods), ii) at room temperature (vs. 60°C–150°C, in solution). Moreover, in contrast to reported solution-based methods that yielded cloudy suspensions of Au(I)–glutathionate, our approach allowed its isolation and in higher amount. The isolated [Au(SG)]_*n*_ complex also offers a novel opportunity for the simple and rapid preparation of ultrasmall oligomeric AuNCs having a size of <2 nm for the gold core, for a possible application in cancer radiotherapy. These oligomeric Au_10–12_(SG)_10–12_ nanoclusters can be obtained by the pH-controlled dissociation of water insoluble [Au(SG)]_*n*_ without involving harmful reducing agents such as CO. Moreover, the isolated luminescent gold(I)–thiolate is also a promising nanomaterial for further pH-controlled reduction-based strategies (e.g., with sodium borohydride) in the development of novel biocompatible classical AuNCs and AuNPs (Yanez-Aulestia et al., 2022).

## Data Availability

The raw data supporting the conclusion of this article will be made available by the authors, without undue reservation.
