# Supplementary material for: The first solid-state route to luminescent Au(I)—glutathionate and its pH-controlled transformation into ultrasmall oligomeric Au10–12(SG)10–12 nanoclusters for application in cancer radiotheraphy
Source: Front Chem. 2023 Jun 5;11:1178225. doi: 10.3389/fchem.2023.1178225 (PMC10277803; doi:10.3389/fchem.2023.1178225)
Supplement: Supplementary file 1 [file DataSheet1.pdf]

Supplementary Material

The first solid-state route to luminescent Au(I)–glutathionate and its pH-controlled transformation into ultrasmall oligomeric $Au_{10–12}(SG)_{10–12}$ nanoclusters for application in cancer radiotheraphy

Andrea Deák,[1*] Pál T. Szabó,[2] Vendula Bednaříková,[3] Jaroslav Cihlář,[3] Attila Demeter,[4] Michaela Remešová,[3] Evelina Colacino[5] and Ladislav Čelko[3]

* **Correspondence:** Andrea Deák: deak.andrea@ttk.hu

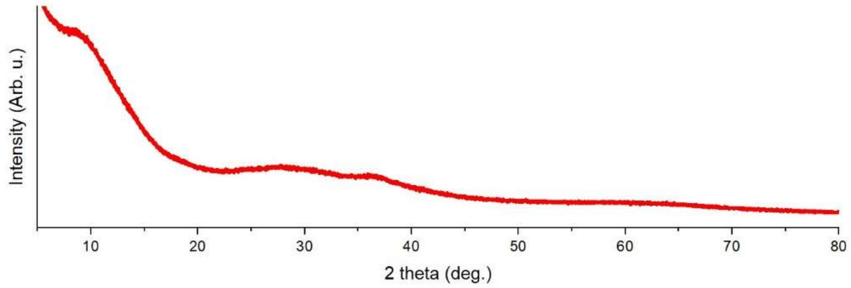

Figure S1. PXRD pattern of the $[Au(SG)]_n$ complex.

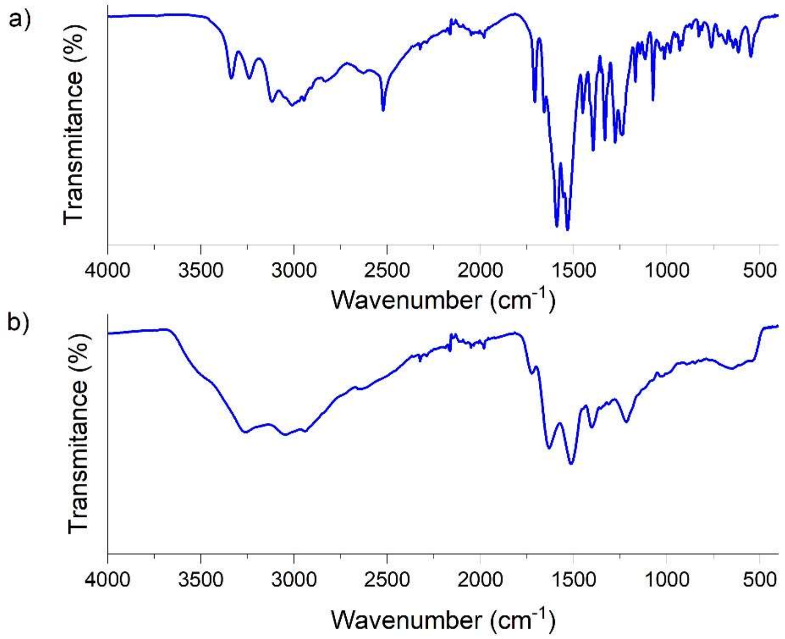

Figure S2. FT-IR spectrum of the a) free GSH ligand and b) its $[Au(SG)]_n$ complex.

The results of simultaneous thermal analysis (STA) are shown in Figures S3 and S4.

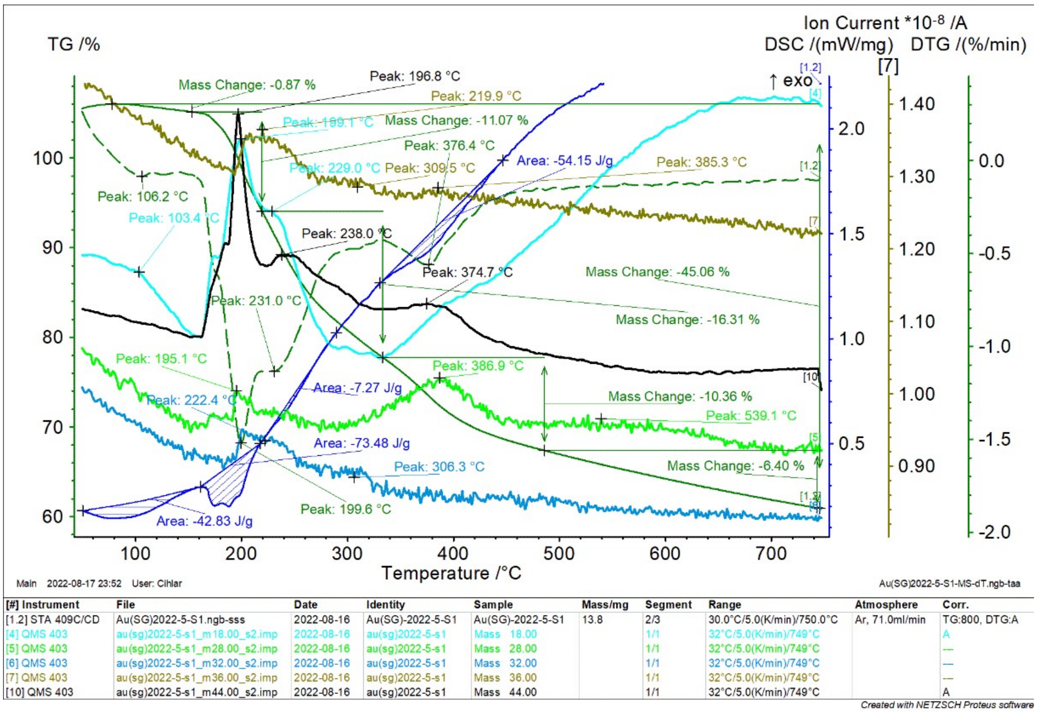

Figure S3. The graph of the STA (TGA-DSC-MS) of the [Au(SG)]$_n$ complex in the temperature range of 30–750 °C.

As shown in Figure S3, the TG curve or its derivative (DTG) showed four peaks (temperature range of 30–500 °C) and a mass loss without a peak for the [Au(SG)]$_n$ complex. The first maximum weight loss of around 100 °C (106.2 °C) was related to the release of physically bound water. The weight of the sample decreased by –0.87%. MS showed only H_2O peaks at 103.4 °C. The second maximum of weight losses around the temperature of 200 °C (199.1 °C) reached –11.07%. These decreases were caused by thermal decomposition of the glutathionate ligands of the [Au(SG)]$_n$ complex, mainly characterized by the release of H_2O (199.1 °C), CO_2 (196.8 °C) and a smaller amount of N_2/C_2H_2 (195.1 °C). The third maximum of weight losses around the temperature of 230 °C (231.0 °C) reached –16.31%. Also, these decreases were due to the continued thermal decomposition of the glutathionate ligands of the [Au(SG)]$_n$ complex, which released mainly H_2O (229.0 °C), CO_2 (238.0 °C). The fourth maximum weight loss in the 327.6 to 376.4 °C temperature range was –10.36%. During this, CO_2 (374.7 °C) and N_2/C_2H_2 residues were released. Peaks of sulphur at 222.4 °C and 306.3 °C and an admixture of HCl in a wide temperature range of 219.9 to 389.2 °C were also detected. Weight losses without a maximum temperature range of 500–750 °C reached – 6.4%. We can assume that by thermal decomposition of the [Au(SG)]$_n$ complex in an inert environment, part of the carbon from the thiolate will remain in the samples. Therefore, after reaching a temperature of 750 °C, the STA program included an additional step, the oxidation of carbon residues in an Ar/O_2 atmosphere at a temperature of 750 °C for 15 minutes. The results in Figure S4 show that the samples contained 19.18% carbon. The total weight loss corresponding to the weight of the thiolate ligands (total weight loss minus the weight loss of water) is 63.32%. Based on the STA results, the calculated Au/SG molar ratio is 1/1.1. This somewhat higher

SG ratio can be related to the presence of residual water (hydrogen bonded to the carboxylic acid group of the glutathionate ligand, released at around 200 °C), whose contribution cannot be excluded from the 63.32% total weight loss.

DSC curves showed the endothermic course of all thermal events in the inert Ar atmosphere and the exothermic course of events in the Ar/O_2 oxidizing atmosphere.

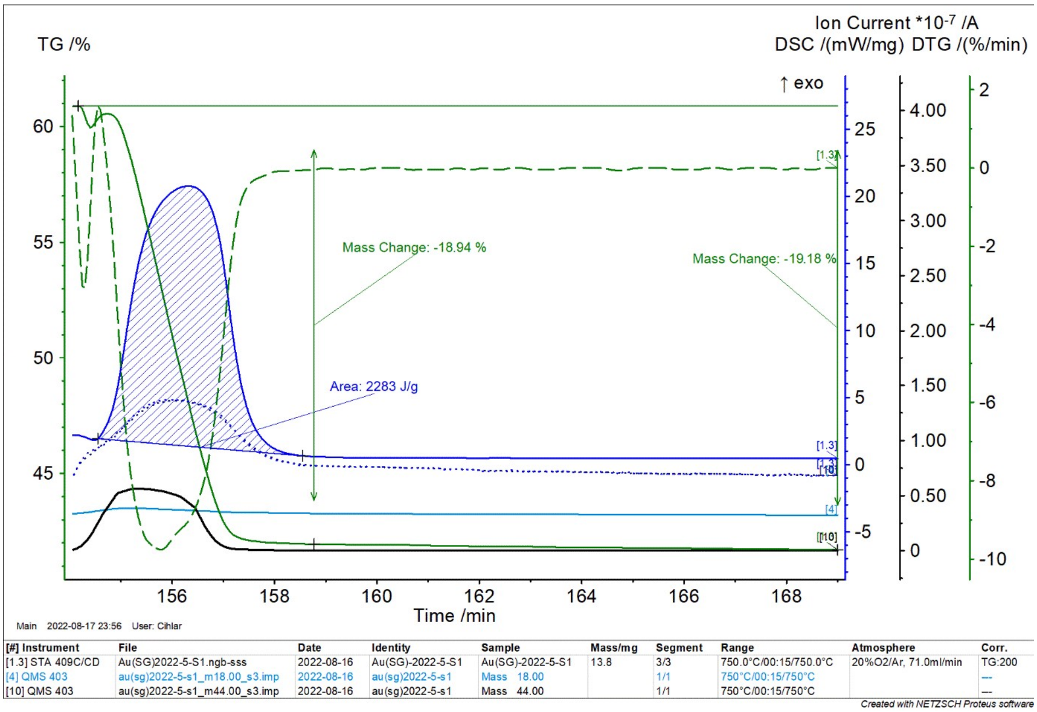

Figure S4. The graph of the STA shows the oxidation of $[Au(SG)]_n$ upon heating in Ar/O_2 atmosphere at a temperature of 750 °C for 15 minutes.

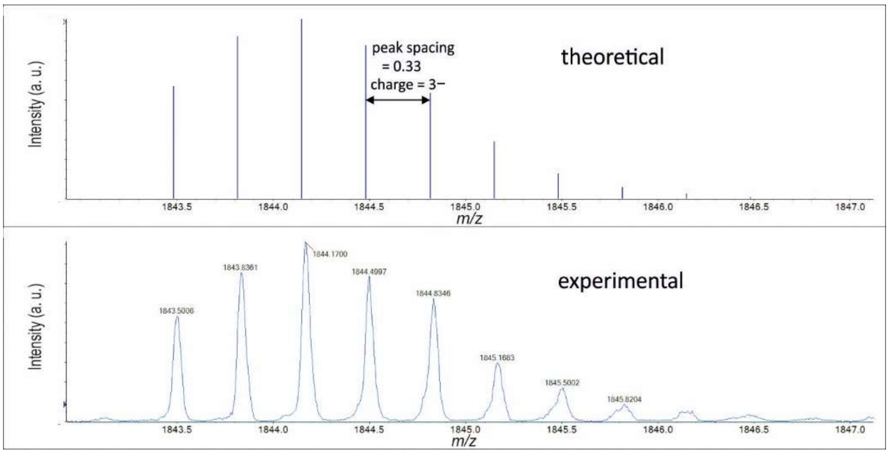

Figure S5. Theoretical (top) and experimental (bottom) isotope pattern of $[Au_{11}(SG)_{11} - 3H^{+}]^{3-}$

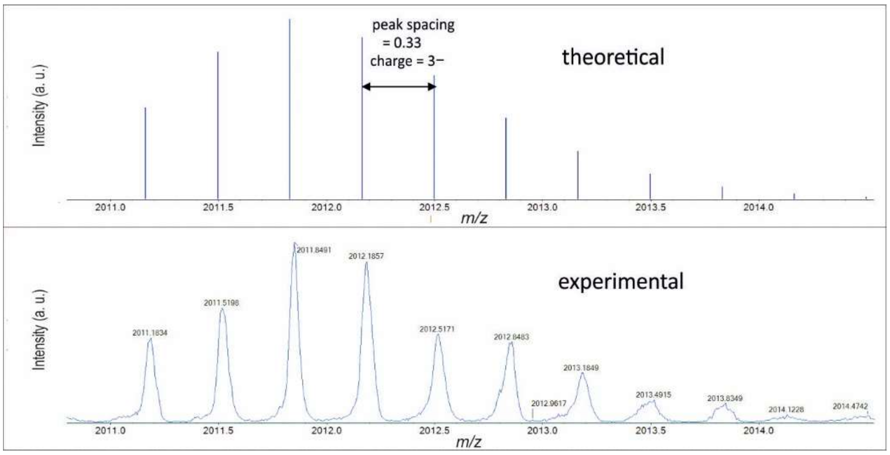

Figure S6. Theoretical (top) and experimental (bottom) isotope pattern of $[Au_{12}(SG)_{12} - 3H^{+}]^{3-}$

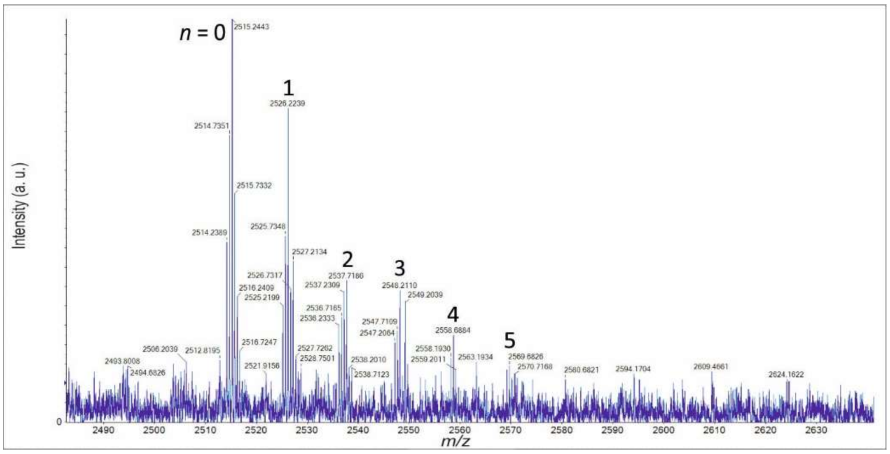

Figure S7. Expanded view of peaks belonging to $[Au_{10}(SG)_{10} - (2+n)H + nNa]^{2-}$.

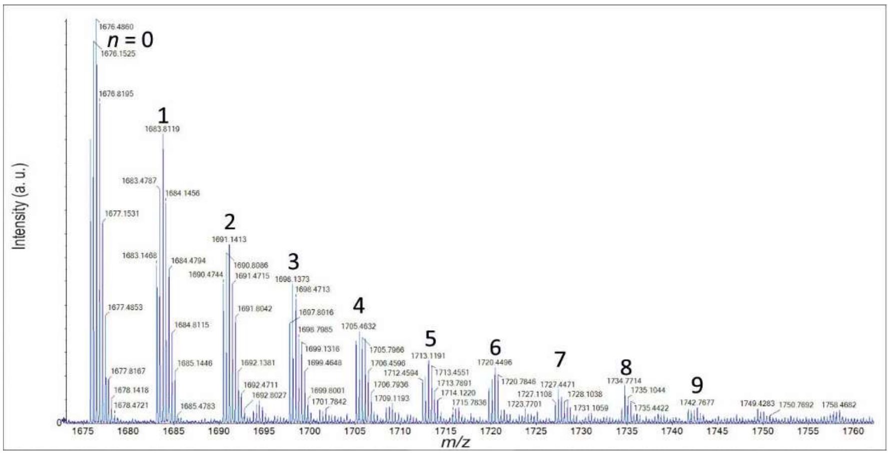

Figure S8. Expanded view of peaks belonging to $[Au_{10}(SG)_{10} - (3+n)H + nNa]^{3-}$.

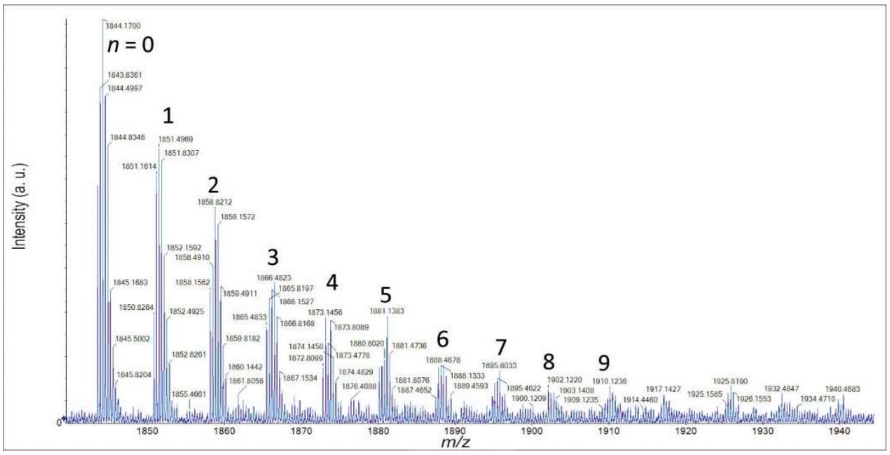

Figure S9. Expanded view of peaks belonging to $[Au_{11}(SG)_{11} - (3+n)H + nNa]^{3-}$.

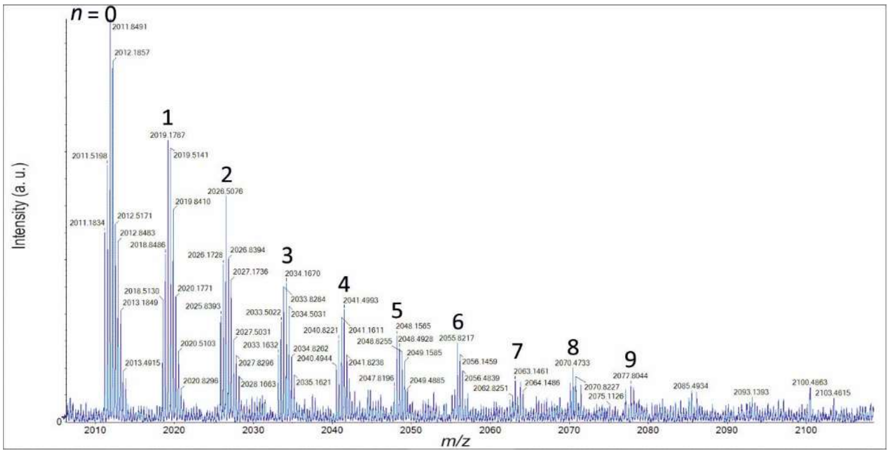

Figure S10. Expanded view of peaks belonging to $[Au_{12}(SG)_{12} - (3+n)H + nNa]^{3-}$.

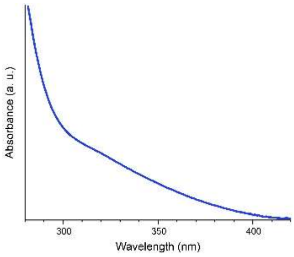

Figure S11. UV-Vis absorption spectrum of Au(I)–glutathionate at neutral pH.

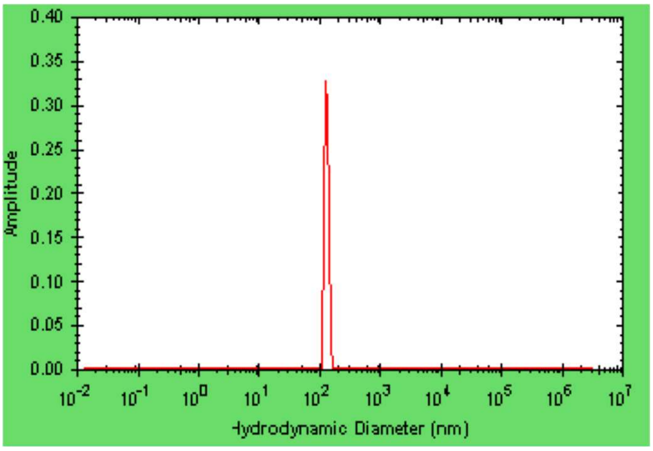

Figure S12. Hydrodynamic diameter of aggregated particles of oligomeric Au(I)–glutathionate NCs measured by DLS.

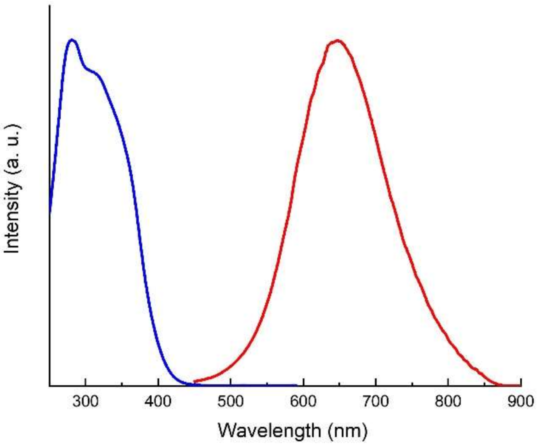

Figure S13. Photoluminescence (red line) and excitation (blue line) spectra of aggregated oligomeric Au(I)–glutathionate NCs.
